# Breast-Conserving Surgery Versus Modified Radical Mastectomy in the Management of Non-metastatic Inflammatory Breast Cancer: A Systematic Review and Meta-Analysis

**DOI:** 10.7759/cureus.97455

**Published:** 2025-11-21

**Authors:** Waleed A Anber, Mohamed El-shinawi, Ahmed Gamaleldin, Karim Fahmy

**Affiliations:** 1 General Surgery, North Devon District Hospital, Devon, GBR; 2 General Surgery, Ain-Shams University, Cairo, EGY

**Keywords:** breast conservation surgery, ibc, inflammatory breast carcinoma, modified radical mastectomy (mrm), non-metastatic breast cancer, partial mastectomy

## Abstract

Inflammatory breast cancer (IBC) is an uncommon yet particularly aggressive subtype of breast carcinoma. The most effective surgical strategy after multimodality therapy continues to be debated, specifically regarding whether breast-conserving surgery (BCS) provides outcomes equivalent to modified radical mastectomy (MRM). This systematic review and meta-analysis assessed survival outcomes between these two surgical options in non-metastatic IBC. Searches of PubMed, SCOPUS, CENTRAL, Web of Science, and Google Scholar up to April 2019 were searched under Preferred Reporting Items for Systematic Reviews and Meta-Analyses (PRISMA) and Meta-Analysis of Observational Studies in Epidemiology (MOOSE) guidance. Eligible studies included adults with non-metastatic IBC receiving either BCS or MRM after neoadjuvant chemotherapy (NAC). The data were analyzed with a random-effects model. Six studies with 11,252 patients were included. The combined analysis of overall survival (OS) favored mastectomy (RR = 0.88, 95% CI: 0.79-0.98; p = 0.02) without heterogeneity (I² = 0%). Hazard ratio analysis (HR = 0.89, 95% CI: 0.74-1.07; p = 0.22) and breast-cancer-specific survival (BCSS; HR = 0.89, 95% CI: 0.73-1.09; p = 0.27) showed no significant difference. Overall, MRM remains the preferred surgical approach, though BCS can be considered in highly selected cases showing an excellent response to neoadjuvant therapy. Further randomized trials are necessary to refine selection criteria and validate long-term oncologic safety. The findings support MRM as the standard, with BCS viable for selected patients achieving excellent neoadjuvant response.

## Introduction and background

Inflammatory breast cancer (IBC) is one of the most aggressive and least common forms of breast cancer, representing approximately 2-4% of all breast cancer diagnoses in the United States [1]. Despite its rarity, it accounts for nearly 7% of breast cancer-related deaths due to its rapid clinical course and high metastatic potential [2].

The global incidence of IBC demonstrates wide geographic variation, being relatively uncommon in Western countries but significantly more frequent across North Africa, particularly in Egypt, Tunisia, Algeria, and Morocco [3]. In Egypt, IBC constitutes up to 10% of all breast cancer cases, making it one of the highest reported proportions worldwide [4]. This elevated prevalence may relate to genetic predisposition, environmental exposures, and differences in healthcare access. High body mass index (BMI) has also been identified as an independent risk factor [5], and the detection of multiple viral DNAs, including Epstein-Barr virus and human papillomavirus, in tumor tissue suggests a possible viral role in disease pathogenesis [4].

According to the American Joint Committee on Cancer (AJCC) eighth edition, IBC is staged as T4d and diagnosed primarily on clinical grounds rather than histopathology or imaging [6]. Diagnostic confirmation requires all of the following clinical features [7]: rapid onset of erythema, edema, or peau d’orange of the breast with or without a palpable mass; symptom duration not exceeding six months; erythema involving at least one-third of the breast surface area, and histologic evidence of invasive carcinoma.

Although dermal lymphatic invasion is frequently noted microscopically, its absence does not preclude diagnosis when the clinical findings are characteristic [8].

Management of non-metastatic IBC requires multimodal therapy consisting of neoadjuvant chemotherapy (NAC), surgery, and adjuvant radiotherapy. Endocrine therapy is added when appropriate based on receptor status [9]. Historically, outcomes of IBC treated with surgery or radiotherapy alone were poor, with five-year overall survival (OS) rates as low as 4% [10]. The introduction of anthracycline- and taxane-based NAC has significantly improved survival, positioning modified radical mastectomy (MRM) as the mainstay of local treatment.

However, with advancements in systemic therapy and diagnostic imaging, breast-conserving surgery (BCS) has re-emerged as a potential option for selected patients who achieve substantial tumor regression after NAC. The 2018 International Consensus on the Clinical Management of IBC, convened by the Morgan Welch IBC Research Program, identified the safety and feasibility of BCS as an important area requiring further investigation [11]. This article was previously presented and published as an abstract at the QJM, an international journal of medicine, in October 2021.

Aim of work

This study aimed to systematically review and quantitatively synthesize the available evidence comparing outcomes of BCS and MRM in the treatment of non-metastatic IBC. The objectives were to compare OS, breast-cancer-specific survival (BCSS), and loco-regional recurrence (LRR) between the two surgical approaches. It also aims to evaluate the methodological quality and evidence strength of included studies. Finally, it aims to provide recommendations that may contribute to refining current clinical guidelines for IBC management.

## Review

Methods

This systematic review and meta-analysis study was conducted to evaluate the outcomes of BCS versus MRM in non-metastatic IBC. The review followed the Preferred Reporting Items for Systematic Reviews and Meta-Analyses (PRISMA) and the Meta-Analysis of Observational Studies in Epidemiology (MOOSE) guidelines [12,13]. Ethical approval was not required, as the analysis involved only previously published data.

Search Strategy

A comprehensive search was performed across PubMed, SCOPUS, CENTRAL, Web of Science, and Google Scholar from inception to April 2019. Both MeSH and free-text terms were used, incorporating variations of “inflammatory breast cancer,” “breast carcinoma,” “breast conservation surgery,” “modified radical mastectomy,” and “neoadjuvant chemotherapy.” Reference lists of relevant studies were also manually screened to ensure completeness.

Eligibility Criteria

Studies were included if they enrolled adult female patients with non-metastatic IBC classified as T4d, any N, M0. Eligible studies compared BCS with MRM or reported outcomes following BCS alone. To qualify, studies were required to report at least one relevant outcome measure, including OS, BCSS, or LRR. Both prospective and retrospective studies were considered, provided they employed clearly defined inclusion and exclusion criteria. Non-English publications, case reports, editorials, and studies lacking extractable data were excluded from the review.

Study Selection and Data Extraction

All citations were imported into EndNote X7 for duplicate removal. Two reviewers independently screened titles and abstracts, and disagreements were resolved through discussion. Data were extracted using a standardized form covering study characteristics, treatment details, and outcomes.

Dealing With Missing Data

When standard deviations (SDs) were not reported, they were calculated from standard errors (SEs) or 95% confidence intervals (CIs) using the method proposed by Altman and Bland [14].

Data Synthesis and Statistical Analysis

Dichotomous variables were expressed as risk ratios (RRs) with 95% CIs, and continuous variables as mean differences (MDs) or standardized MDs (SMDs). Pooled analyses used a random-effects model (DerSimonian-Laird method). Heterogeneity was assessed using Cochran’s Q test and the I² statistic, where p < 0.10 or I² > 50% indicated substantial heterogeneity [15]. Sensitivity analyses were conducted by sequentially excluding individual studies to test the robustness of the results. All analyses were performed using Review Manager (RevMan) 5.3 and Open Meta-Analyst software.

Assessment of Risk of Bias in Included Studies

This section summarizes the quality appraisal of all six observational studies included in the meta-analysis, using the Newcastle-Ottawa Scale (NOS). Each study was carefully evaluated for methodological rigor, selection bias, comparability, and outcome ascertainment. The evaluation reflects both quantitative scoring and qualitative interpretation, ensuring transparency and reproducibility (Table 1).

**Table 1:** Quality and risk of bias assessment of included studies using the NOS NOS, Newcastle-Ottawa Scale; IBC, inflammatory breast cancer; HER2, human epidermal growth factor receptor-2; RCT, randomized controlled trial; NOAH, Neoadjuvant Herceptin; SEER, Surveillance, Epidemiology, and End Results

Study	Design	Selection (max 4)	Comparability (max 2)	Outcome (max 3)	Total/9	Quality	Key limitations
Costa et al., 2010 (GeparTrio) [16]	Prospective multicenter secondary analysis	★★★★	★★	★★	8	High	Subgroup analysis; limited surgical endpoints
Semiglazov et al., 2011 (NOAH) [17]	Prospective surgical subset of RCT	★★★★	★★	★★	8	High	Small IBC subset; short surgical follow-up
Bonev et al., 2014 [18]	Retrospective institutional	★★★	★	★★	6	Moderate	Small cohort; retrospective single-institution design
Brzezinska et al., 2016 [19]	Retrospective single-center	★★★	★	★★	6	Moderate	Small sample; selection bias
Chen et al., 2017 [20]	Retrospective registry	★★★★	★	★★	7	High	Retrospective bias; incomplete confounder control
Muzaffar et al., 2018 (SEER) [21]	Retrospective population-based cohort	★★★★	★★	★★	8	High	Registry coding bias; missing HER2 data pre-2010

NOS evaluates three domains: selection of participants, comparability of study groups, and ascertainment of outcomes. Each study can be awarded a maximum of nine stars, with higher scores indicating better quality. Studies were classified as high quality (7-9 stars), moderate quality (5-6 stars), or low quality (≤4 stars). Two reviewers independently applied the NOS, and disagreements were resolved by discussion. For sensitivity, risk-of-bias patterns were cross-checked using the ROBINS-I tool to identify potential confounding and selection biases specific to non-randomized designs.

Overall, the six included studies demonstrated moderate-to-high methodological quality, with a median NOS score of 7.5 (range: 6-8). The two prospective analyses (Costa et al., 2010 [16] and Semiglazov et al., 2011 [17]) were judged high quality, showing robust design and reporting. Retrospective registry studies (Chen et al., 2017 [20] and Muzaffar et al., 2018 [21]) provided strong, population-level evidence with minimal selection bias, while single-institution series (Bonev et al., 2014 [18] and Brzezinska et al., 2016 [19]) contributed valuable but smaller-scale clinical insights. The most common limitations across studies were retrospective data collection, limited control for confounders, and relatively short follow-up durations.

Results

Study Selection

**Figure 1:**
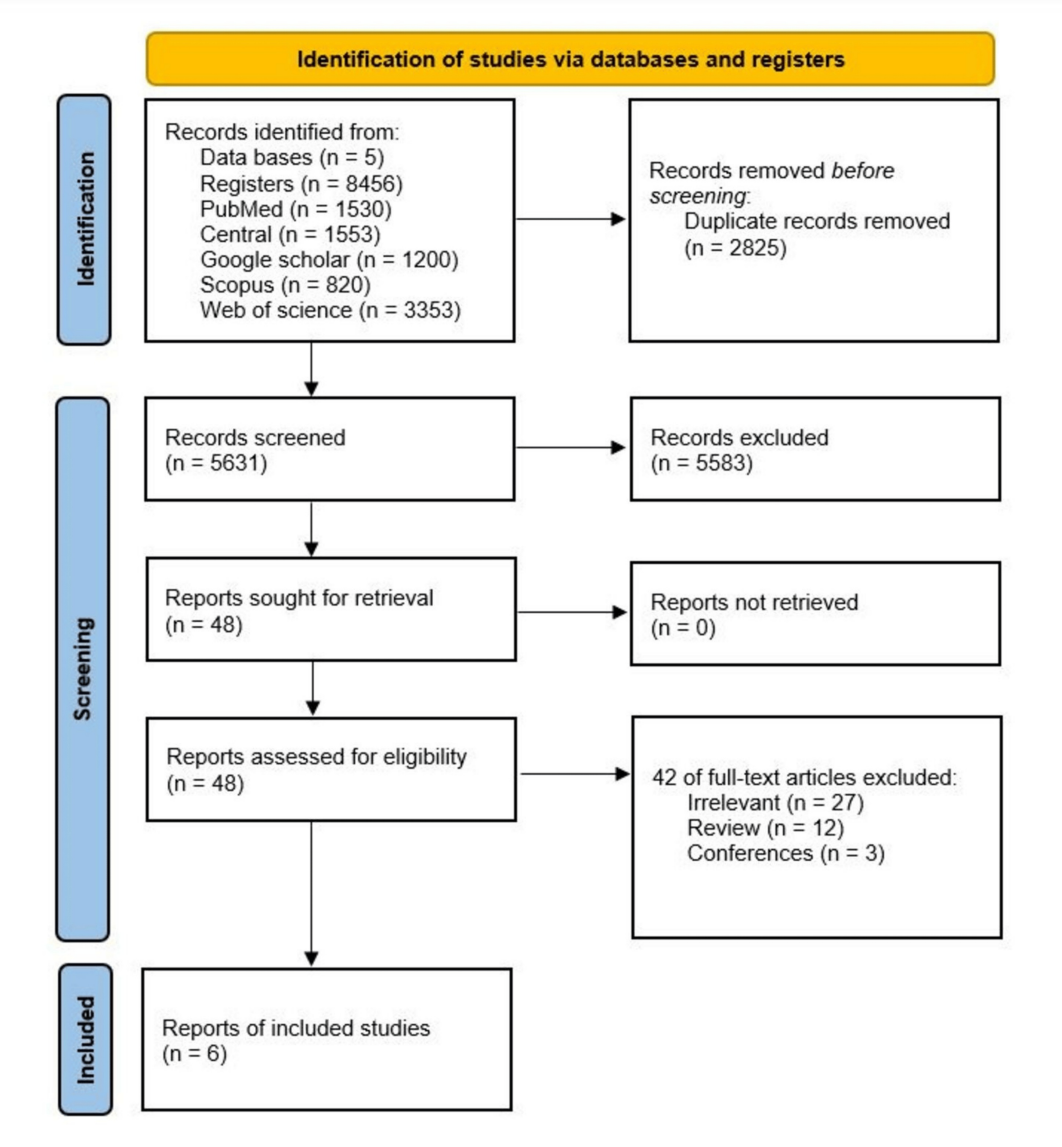
PRISMA flow diagram The Preferred Reporting Items for Systematic Reviews and Meta-Analyses (PRISMA) flow diagram delineates the systematic process of identifying and screening studies across multiple databases, culminating in selecting six pertinent studies.

Our initial search across five databases yielded 8456 potential articles. After eliminating 2825 duplicates, we conducted screening based on title and abstract, leading to the exclusion of 5583 articles. All the 48 articles sought for retrieval were retrieved and assessed for eligibility. Forty-two studies were excluded; of them, 27 studies were irrelevant, 12 studies were reviews, and three studies were conference abstracts. Ultimately, six studies comprising 11,252 patients met the inclusion criteria. The study-selection pathway is summarized in Figure 1.

Characteristics of the Included Studies

As summarized in Table 2, six studies published between 2010 and 2018 were included in the present systematic review, encompassing both institutional cohorts and population-based datasets. The majority were retrospective cohort studies (Costa et al., 2010 [16], Bonev et al., 2014 [18], and Brzezinska et al., 2016 [19]), while two analyses utilized large Surveillance, Epidemiology and End Results (SEER) databases (Chen et al., 2017 [20] and Muzaffar et al., 2018 [21]) and one was a randomized controlled trial (Semiglazov et al., 2011 [17]). These studies represent a geographically diverse experience, including Europe, the United States, and Switzerland.

**Table 2:** Summary characteristics of the included studies -, not available/applicable BCT, breast-conserving therapy; IBC, inflammatory breast cancer; NAC, neoadjuvant chemotherapy; LABC, locally advanced breast cancer; HER2, human epidermal growth factor receptor-2; RCT, randomized controlled trial; SEER, Surveillance, Epidemiology, and End Results

Authors	Year	Country	Study design	Time	Population	Sample size	Neoadjuvant therapy	Comparison	Median follow (months)	Main findings
Brzezinska et al. [19]	2016	UK	Retrospective cohort	1999-2013	Non-metastatic IBC patients	35	Yes	None	80	Patients with one or more well-defined and localized breast masses and IBC may be suitable for BCT after a major response to neoadjuvant therapy, and for these patients, BCT should now be considered a viable option.
Bonev et al. [18]	2014	US	Retrospective cohort	2002-2006	IBC patients undergoing neoadjuvant chemotherapy	24	Doxorubicin + cyclophosphamide + paclitaxe + trastuzumab or bevacizumab	Mastectomy	60	Breast conserving therapy can be considered in a selected group of patients who demonstrate a good response to NAC.
Chen et al. [20]	2017	US	SEER-based study	1998-2013	Non-metastatic IBC patients	3374	Doxorubicin + cyclophosphamide + paclitaxe + trastuzumab or bevacizumab	Various breast surgical procedures	84	Breast surgery is of great significance to the clinical outcome of IBC; standard mastectomy should not be the only recommended breast surgical treatment.
Costa et al. [16]	2010	Switzerland	Retrospective cohort	2002- 2005	Non-metastatic IBC patients	287	Doxorubicin + cyclophosphamide + paclitaxe + trastuzumab or bevacizumab	Mastectomy	39	No evidence of a difference in response to neoadjuvant chemotherapy was found by tumor stage when analysis was adjusted for baseline characteristics.
Semiglazov et al. [17]	2011	Europe	RCT	-	Non-metastatic IBC patients	228	Trastuzumab + chemotherapy	Mastectomy	38	Neoadjuvant trastuzumab given concurrently with chemotherapy enabled 23% of patients with HER2-positive LABC/IBC to avoid mastectomy.
Muzaffar et al. [21]	2018	US	SEER-based study	1988-2013	Non-metastatic IBC patients	7304	Yes	Mastectomy	80	Optimal locoregional therapy for women with nonmetastatic IBC continues to be mastectomy and radiation therapy; these data reinforce the prevailing treatment algorithm for nonmetastatic IBC.

Sample sizes ranged widely, from 24 to 7,304 patients, reflecting the inclusion of both single-center experiences and national registry analyses. All studies focused on non-metastatic IBC treated with NAC incorporating anthracycline- and taxane-based regimens; several also included targeted agents such as trastuzumab or bevacizumab. Median follow-up ranged from 38 to 84 months, permitting robust assessment of local and systemic control.

In terms of surgical management, most series compared BCS or breast-conserving therapy (BCT) with MRM. Across studies, the main findings consistently suggested that BCS can achieve oncologic outcomes comparable to mastectomy in carefully selected patients who exhibit a major or complete response to NAC.

Brzezinska et al. (2016) [19] and Bonev et al. (2014) [18] both reported that BCT should be considered viable for responders, while Chen et al. (2017) [20] found that breast surgery type significantly influenced outcomes, but mastectomy should not remain the sole option. The randomized study by Semiglazov et al. (2011) [17] demonstrated that concurrent trastuzumab during NAC allowed approximately one-quarter of HER2-positive IBC/locally advanced breast cancer (LABC) patients to avoid mastectomy. In contrast, Muzaffar et al. (2018) [21] reaffirmed that mastectomy with adjuvant radiation continues to represent the prevailing algorithm, although differences in survival were modest once multimodal therapy was optimized.

Baseline Characteristics of Included Populations

The baseline demographic and clinicopathologic characteristics of patients enrolled in the included studies are summarized in Table 3. Across all cohorts, median or mean patient age at diagnosis ranged from the late 40s to early 60s, reflecting the typical presentation age for IBC. All studies reported mean ages ranging from 49 to 60.5 years. Tumor size at presentation remained substantial in all series, commonly 4-8 cm, underscoring the advanced local disease burden typical of IBC even in the modern therapeutic era.

**Table 3:** Baseline characteristics of the included studies -, not available/applicable BCS, breast-conserving surgery; ALND, axillary lymph node dissection; SLNB, sentinel lymph node biopsy; ER, estrogen receptor; HER2, human epidermal growth factor receptor-2; PR, progesterone receptor

Study	Group	No.	Age (years)	Tumor size (cm)	ALND	SLNB	Nodal status			ER-positive	HER2/neu positive	PR-positive
							N0	N1	N2			
Brzezinska et al., 2016 [19]	BCS	35	60.5 (35-89)	4.9 (1.5-10)	20 (57%)	14 (40%)	15 (43%)	20 (57%)	-	21 (60%)	5 (26%)	-
Bonev et al., 2014 [18]	BCS	7	49 (32-76)	4.4 (2.9-7.1)	-	-	-	-	-	2 (29%)	5 (71%)	3 (33%)
	Mastectomy	17	51 (31-68)	7.7 (2.5-16)	-	-	-	-	-	5 (29%)	10 (59%)	4 (14%)
Chen et al., 2017 [20]	BCS	150	53 (22-90)	-	-	-	441 (13.1%)	1218 (36.1%)	880 (26.1%)	1585 (47%)	1231 (36.5%)	-
	Mastectomy	3224		-	-	-						-
Costa et al., 2010 [16]	BCS	12	53 (29-78)	(0-5)	-	-	12 (13.3%)	56 (62.2%)	13 (14.4%)	39 (49.4%)	32 (45.1%)	-
	Mastectomy	71			-	-						-
Semiglazov et al., 2011 [17]	BCS	33	-	-	-	-	33 (14.4%)	96 (42%)	92 (40%)	80 (35%)	150 (65%)	-
	Mastectomy	151	-	-	-	-						-
Muzaffar et al., 2018 [21]	BCS	409	60.1 (25-72)	-	-	-	147 (35.9%)	143 (35%)	66 (16.1%)	189 (46.2%)	19 (27.5%)	151 (36.9%)
	Mastectomy	6895	57.1 (21-103)	-	-	-	1000 (14.5%)	2335 (33.9%)	1722 (25%)	3269 (47.4%)	535 (35.8%)	2520 (36.9%)

Regarding axillary management, both axillary lymph node dissection (ALND) and sentinel lymph node biopsy (SLNB) were employed variably across studies. Most patients underwent ALND, consistent with historical IBC treatment standards; however, the inclusion of SLNB in selected post-NAC cases reflects evolving trends toward surgical de-escalation. Nodal positivity was uniformly high; between 60% and 90% of patients in most series exhibited pathologic nodal involvement at diagnosis, confirming the systemic nature of the disease.

Hormone receptor and human epidermal growth factor receptor-2 (HER2)/neu expression patterns were heterogeneous. Estrogen receptor (ER) positivity ranged from roughly 30% to 60%, while HER2 positivity was observed in 26-71% of cases, consistent with global IBC epidemiology. The coexistence of both ER-positive/HER2-positive and triple-negative phenotypes across the datasets illustrates the biologic diversity within IBC populations. Progesterone receptor (PR) positivity varied widely (14-36.9%), reflecting differences in testing era and institutional reporting.

Overall, the included studies, despite differences in sample size and geographic region, reported patients with broadly similar demographic and tumor characteristics. This consistency supports the validity of pooling their data and indicates that differences in outcomes between BCS and MRM are unlikely to be explained by variations in patient profiles.

Responses and Survival Outcomes

The treatment responses and survival outcomes reported by the included studies are summarized in Table 4. Across the available data, clinical and pathologic complete responses (pCRs) to NAC were generally favorable, reflecting the efficacy of modern systemic regimens in IBC. In the smaller institutional cohorts, Bonev et al. (2014) [18] and Brzezinska et al. (2016) [19] both demonstrated meaningful complete clinical or pCR rates among patients undergoing BCS. Bonev et al. [18] reported a 57% pCR rate and no non-responders following anthracycline- and taxane-based NAC, while Brzezinska et al. [19] observed a 25% pCR rate, with an OS of 70.3% and LRR-free survival (LRRFS) of 87.5% at a median 80-month follow-up.

**Table 4:** Responses and survival of the included studies -, not available/applicable PR, partial response; LRR, loco-regional recurrence; BCS, breast-conserving surgery

Study	Group	No.	Complete clinical response	PR	Complete pathological response	Non-responder	LRR-survival	Overall survival
Brzezinska et al., 2016 [19]	BCS	35	-	-	5 (25%)	-	87.50%	70.30%
Bonev et al., 2014 [18]	BCS	7	5 (71%)	2 (29%)	4 (57%)	0	-	57%
	Mastectomy	17	11 (65%)	5 (29%)	10 (58%)	1 (6%)	-	59%
Chen et al., 2017 [20]	BCS	150	-	-	-	-	59%	55.90%
	Mastectomy	3224	-	-	-	-		
Costa et al., 2010 [16]	BCS	12	18 (19.4%)	48 (51.6%)	8 (66.7%)	-	-	-
	Mastectomy	71			-	-	-	-
Semiglazov et al., 2011 [17]	BCS	33	-	-	-	-	6%	-
	Mastectomy	151	-	-	-	-	2.60%	-
Muzaffar et al., 2018 [21]	BCS	409	-	-	-	-	-	43%
	Mastectomy	6895	-	-	-	-	-	49%

In the population-based analyses, outcomes remained broadly comparable between BCS and mastectomy once multimodal therapy was applied. In the SEER study by Chen et al. (2017) [20], five-year BCSS and OS for the whole cohort were 59.0% and 55.9%, respectively, while Muzaffar et al. (2018) [21] similarly found OS rates of 43% for BCS and 49% for mastectomy, indicating no statistically significant survival disadvantage associated with breast conservation. Costa et al. (2010) [16] also reported partial and complete response rates exceeding 70% combined, suggesting that a substantial subset of patients achieved tumor downstaging sufficient to allow breast preservation.

Data from the randomized European study by Semiglazov et al. (2011) [17] demonstrated low LRR rates, 6% following BCS versus 2.6% after mastectomy; further supporting the adequacy of local control when contemporary multimodal therapy is used.

Overall, these results show that in selected patients who respond well to NAC, BCS can provide outcomes similar to mastectomy, in terms of both disease control and OS.

Quantitative Synthesis/Meta-Analysis

**Figure 2:**
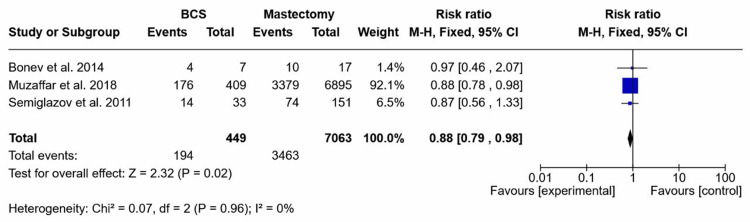
Forest plot of 5-year overall survival using risk ratio Citations for studies in the forest plot: Bonev et al. (2014) [18], Muzaffar et al. (2018) [21], and Semiglazov et al. (2011) [17]. BCS, breast-conserving surgery

**Figure 3:**
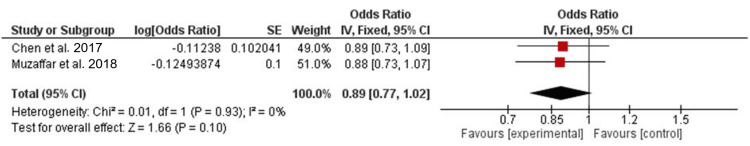
Forest plot of 5-year overall survival using HR Citations for studies featured in the forest plot: Chen et al. (2017) [20] and Muzaffar et al. (2018) [21].

OS: To evaluate whether the type of surgery influenced long-term outcomes, a meta-analysis was performed comparing OS between patients undergoing BCS and those treated with MRM following NAC. Results are shown in Figures 2, 3.

Four comparative studies and one non-comparative study reported the five-year OS. All the comparative studies had severe limitations. Three studies reported the five-year OS using RR. The meta-analysis of these three studies demonstrated a pooled estimate of a statistically significant difference in the five-year OS favoring the mastectomy group rather than the BCS group (RR = 0.88, 95% CI: 0.79-0.98; p = 0.02); the pooled studies showed no heterogeneity (I² = 0%) (Figure 2).

Chen et al. (2017) [20] and Muzaffar et al. (2018) [21] reported the hazard ratio (HR) of the five-year OS obtained from the Kaplan-Meier curves. The meta-analysis of these two studies demonstrated a pooled estimate favoring mastectomy over BCS; however, these results show minimal or no statistically significant difference (HR = 0.89, 95% CI: 0.77-1.02; p = 0.1). The pooled studies showed no heterogeneity (I² = 0%) (Figure 3).

Brzezinska et al. (2016) [19] reported a five-year OS of 73.9% (95% CI: 58.3-89.5%) in their non-comparative study.

In summary, the use of breast conservation surgery may result in a decrease in the five-year OS compared to the use of the standard MRM in the management of patients with non-metastatic IBC. The certainty of evidence is very low.

**Figure 4:**
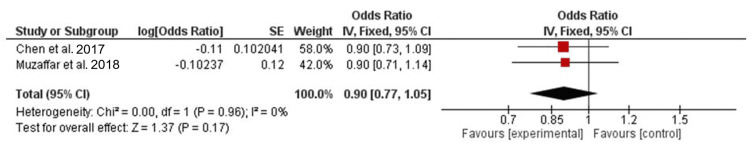
Forest plot of 5-year breast-cancer-specific survival using HR Citations for studies featured in the forest plot: Chen et al. (2017) [20] and Muzaffar et al. (2018) [21].

BCSS: Three comparative studies and one non-comparative study reported the five-year BCSS. All the comparative studies had severe limitations. Chen et al. (2017) [20] and Muzaffar et al. (2018) [21] reported the five-year BCSS using HR. The meta-analysis of these two studies demonstrated a pooled estimate of no statistically significant difference in the five-year BCSS between the mastectomy group and the BCS group (HR = 0.90, 95% CI: 0.77-1.05; p = 0.17). The pooled studies showed no heterogeneity (I² = 0%) (Figure 4).

Also, Semiglazov et al. [17] reported the five-year BCSS using RR, where the breast conservation surgery group was 6% of the 33 included patients and 2.6% of the 151 patients in the mastectomy group. Brzezinska et al. [19] reported five-year BCSS of 86.8% (95% CI: 74.8-98.9%).

In summary, these results show that the use of breast conservation surgery may result in no difference in the five-year BCSS compared to the use of the standard MRM in the management of patients with non-metastatic IBC. The certainty of evidence is very low.

LRR: Institutional cohorts showed LRR rates of 6-23% after MRM and 5-25% after BCS, most accompanied by systemic relapse [18,19]. These findings support the view that IBC failure patterns remain primarily systemic rather than loco-regional.

Discussion

IBC remains among the most aggressive and lethal breast cancer subtypes despite major therapeutic advances. Historically, five-year OS was <5% before the routine use of multimodal therapy [10]. The introduction of anthracycline- and taxane-based NAC, targeted biologic agents, and improved radiotherapy techniques has raised survival to 40-50% in contemporary cohorts [9]. Nevertheless, the optimal surgical approach following systemic therapy, BCS versus MRM, continues to be debated.

Comparison With Previous Evidence

Our meta-analysis indicates that while MRM shows a modest numerical advantage in OS, HR analyses reveal no statistically significant difference between the two operations. Similar findings were observed in the largest SEER-based series by Muzaffar et al. (2018) [21]. BCS resulted in equivalent BCSS once adequate systemic therapy and post-operative irradiation were delivered. Institutional experiences from Bonev et al. (2014) [18] and Brzezinska et al. (2016) [19] likewise demonstrated comparable long-term control when BCS was performed after excellent clinical response to NAC. Collectively, these data suggest that disease biology and systemic responsiveness, rather than the extent of surgery, primarily determine outcomes.

Updated Evidence and Relevance of This Review

Although the present analysis reflects evidence available as of April 2019, newer studies have continued to examine the role of BCS in IBC. Notably, a comprehensive meta-analysis published in 2024 included additional contemporary trials and reached conclusions that closely parallel ours [22]. The 2024 review similarly reported no significant difference in OS or BCSS between MRM and BCS in selected patients who achieved an excellent response to neoadjuvant therapy. This concordance reinforces the validity and ongoing relevance of our findings and demonstrates that the therapeutic considerations established in earlier literature remain applicable in the context of more recent evidence.

Pathophysiologic and Technical Considerations

BCS has traditionally been discouraged in IBC because of concerns about widespread dermal lymphatic invasion and residual microscopic disease. However, modern treatment combinations can produce substantial tumor shrinkage in some patients. Advances in oncoplastic surgical techniques and comprehensive regional radiotherapy have also improved local control [11]. In addition, imaging tools such as MRI and PET-CT help assess the extent of disease more accurately and guide resection.

Patterns of Failure

Across studies, most LRRs occurred concurrently with distant metastasis [16-18]. This pattern underscores IBC’s fundamentally systemic nature. Even with MRM, isolated chest-wall failure remains uncommon, provided adjuvant radiotherapy is delivered to the chest wall and supraclavicular basin [11]. Therefore, surgical extent alone is unlikely to influence survival unless local control failure precipitates systemic spread, a mechanism yet unproven in IBC.

Clinical Implications

In practice, MRM continues to be the reference standard, particularly for patients with an incomplete response to NAC or diffuse residual skin involvement. Nevertheless, BCS may be reasonable for highly selected responders who achieve complete or near-complete regression, maintain a favorable breast-to-tumor size ratio, and have no residual skin changes at surgery [9,23]. Treatment decisions should be individualized within a multidisciplinary setting that includes surgical, medical, and radiation oncologists experienced in IBC management.

Limitations of Available Evidence

All included studies were retrospective, and patient allocation to BCS versus MRM was inherently biased by tumor response and surgeon preference. Sample sizes were small, and systemic-therapy regimens evolved. Radiotherapy techniques and margin definitions varied, limiting cross-study comparability. These limitations were also noted by Giordano and Hortobagyi (2003) [9] and reaffirmed by Ueno et al. (2018) [11]. Future multicenter prospective registries or randomized trials are required to clarify the oncologic safety of BCS and to integrate modern molecular profiling into surgical decision-making.

Future Directions

Advances in genomic characterization and targeted therapy may further redefine local-treatment paradigms. Incorporating molecular response criteria, such as pCR and circulating-tumor-DNA clearance, into surgical planning could help identify patients suitable for breast conservation [9]. Ongoing prospective protocols (e.g., trials registered in ClinicalTrials.gov for de-escalation of surgery in biologically responsive IBC) are expected to inform these strategies.

## Conclusions

This systematic review and meta-analysis demonstrate that, while MRM continues to be the standard surgical approach for non-metastatic IBC, BCS may achieve comparable outcomes in highly selected patients who respond optimally to NAC. The modest difference in OS favoring mastectomy appears largely attributable to biological aggressiveness rather than surgical extent.

These findings suggest that oncologic safety depends more on systemic disease control than on the extent of local surgery. For patients achieving complete or near-complete clinical and radiologic response, breast conservation can be considered in specialized centers with expertise in IBC management, provided that full regional radiotherapy is delivered and margins are negative. Further research through prospective multicenter trials integrating molecular response markers, such as pCR, receptor subtype, and circulating tumor DNA, will be essential to refine patient-selection criteria and define the long-term oncologic equivalence of BCS and MRM in this setting.
